# Online singing interventions for postnatal depression in times of social isolation: a feasibility study protocol for the SHAPER-PNDO single-arm trial

**DOI:** 10.1186/s40814-022-01112-1

**Published:** 2022-07-18

**Authors:** Rebecca H. Bind, Carolina Estevao, Daisy Fancourt, Katie Hazelgrove, Kristi Sawyer, Lavinia Rebecchini, Celeste Miller, Paola Dazzan, Nick Sevdalis, Anthony Woods, Nikki Crane, Manonmani Manoharan, Alexandra Burton, Hannah Dye, Tim Osborn, Lorna Greenwood, Ioannis Bakolis, Maria Baldellou Lopez, Rachel Davis, Rosie Perkins, Carmine M. Pariante

**Affiliations:** 1 Department of Psychological Medicine, Institute of Psychiatry, Psychology and Neuroscience, King’s College London, 5 Cutcombe Rd, London, SE5 9RT UK; 2 Department of Behavioural Science and Health, University College London, Gower Street, London, WC1E 6BT UK; 3 Centre for Implementation Science, Health Service and Population Research Department, Institute of Psychiatry, Psychology & Neuroscience, 16 De Crespigny Park, London, SE5 8AB UK; 4 Culture Team, King’s College London, Somerset House East Wing, Strand, WC2R 2LS UK; 5 South London and Maudsley NHS Foundation Trust, Denmark Hill, London, SE5 8AZ UK; 6 Breathe Arts Health Research, The Clarence Centre, 6 St George’s Circus, London, SE1 6FE UK; 7 Centre for Performance Science, Royal College of Music, London, UK; 8 Faculty of Medicine, Imperial College London, London, UK

**Keywords:** Arts intervention, Online delivery, Postnatal depression, Mental health, Singing, COVID-19

## Abstract

**Background:**

Postnatal depression (PND) affects 13% of new mothers, with numbers rising during the COVID-19 pandemic. Despite this prevalence, many women have difficulty with or hesitancy towards accessing pharmacological and/or psychological interventions. Group-based mother-baby activities, however, have a good uptake, with singing improving maternal mental health and the mother-infant relationship. The recent lockdowns highlight the importance of adapting activities to an online platform that is wide-reaching and accessible.

**Aims:**

The SHAPER-PNDO study will primarily analyse the feasibility of a 6-week online singing intervention, Melodies for Mums (M4M), for mothers with PND who are experiencing barriers to treatment. The secondary aim of the SHAPER-PNDO study will be to analyse the clinical efficacy of the 6-week M4M intervention for symptoms of postnatal depression.

**Methods:**

A total of 120 mothers and their babies will be recruited for this single-arm study. All dyads will attend 6 weekly online singing sessions, facilitated by Breathe Arts Health Research. Assessments will be conducted on Zoom at baseline and week 6, with follow-ups at weeks 16 and 32, and will contain interviews for demographics, mental health, and social circumstances, and biological samples will be taken for stress markers. Qualitative interviews will be undertaken to understand the experiences of women attending the sessions and the facilitators delivering them. Finally, data will be collected on recruitment, study uptake and attendance of the programme, participant retention, and acceptability of the intervention.

**Discussion:**

The SHAPER-PNDO study will focus on the feasibility, alongside the clinical efficacy, of an online delivery of M4M, available to all mothers with PND. We hope to provide a more accessible, effective treatment option for mothers with PND that can be available both during and outside of the pandemic for mothers who would otherwise struggle to attend in-person sessions, as well as to prepare for a subsequent hybrid RCT.

**Trial registration:**

ClinicalTrials.gov Identifier: NCT04857593. Registered retrospectively on 22 April 2021. The first participants were recruited on 27 January 2021, and the trial is ongoing.

**Supplementary Information:**

The online version contains supplementary material available at 10.1186/s40814-022-01112-1.

## Key messages regarding feasibility

### What uncertainties exist regarding the feasibility?

The feasibility of using an online singing intervention for new mothers with postnatal depression and their babies is currently unknown. In addition, the feasibility of utilising this intervention not only in times of social isolation, such as during a pandemic, but thereafter for mothers with additional barriers to treatment, is unknown, in particular, recruitment rates, retention rates, study completion rates, acceptability of intervention, and scalability to power a subsequent RCT.

## Introduction

### Background

#### Postnatal depression

Postnatal depression (PND) is an increasingly common mental health disorder, affecting up to 20% of new mothers, and typically manifests as low mood, fatigue, anhedonia, insomnia and irritability, and in severe cases, suicidal thoughts, and gestures [1, 2]. Given that lack of social support is a well-known risk factor for the development of PND [3, 4], it is unsurprising that recent meta-analyses have identified significant rises in depression and anxiety in pregnant and postnatal women since the COVID-19 pandemic began [5, 6]. The combination of prolonged periods of social isolation, coupled with increases in domestic violence [7], unprecedented social, psychological, and financial stress, and having to adapt to an unexpected childrearing process [5], has put new mothers at much greater risk of becoming unwell during the pandemic. As such, women’s mental health during the transition to motherhood must be even more closely monitored and interventions made readily accessible, especially given that depression in the perinatal period not only affects mothers themselves, but can also lead to long-term difficulties in the mother-infant relationship [8] and can contribute to poorer offspring outcomes [9].

#### Barriers to treatment

Even outside of pandemic times, many studies find that access to adequate mental health care during the perinatal period is of grave concern. As we have extensively discussed before (see Fancourt & Perkins (2019)), one of the major challenges in treating PND is that there is still no comprehensive treatment solution for PND that is suitable for all cases, let alone during social isolation and a pandemic. Although pharmacological treatments have promising results, these are often hindered by low uptake and adherence amongst new mothers, while psychotherapy also has inconsistent outcomes and faces similar challenges around uptake and timely treatment [2, 10–13].

Furthermore, in a systematic review investigating barriers to treatment in the perinatal period, Smith et al. [14] identified that barriers to care exist across four separate domains: at the individual level, suggesting a lack of awareness around symptoms and/or services, the pressure of societal stigma, or fear of repercussion, i.e. separation of the baby from the mother; at the health service level, suggesting inadequate healthcare pathways to promptly identify symptoms and appropriately treat; at the sociocultural level, suggesting insufficient resources in place for mothers with language and/or cultural barriers; and finally, at the structural levels, suggesting a lack of cohesive policy in place for healthcare professionals to employ screening and diagnostic tools. Moreover, women with postnatal depression more often than not face additional difficulties (e.g. medical comorbidities, socioeconomic challenges, lack of support) [8], thus rendering treatment access even more challenging, and highlighting the urgency for new treatment options, especially during a pandemic when treatment accessibility is even more scarce.

#### Breathe Melodies for Mums

In non-pandemic times, many mothers engage in community group activities with their babies, such as attending mother-infant play groups. Such activities have been identified as ways of relaxing mothers, creating the opportunity for interaction with other new mothers and their babies, adding variation to each day, and also providing mothers with a sense of personal fulfilment [10, 15].

Recent studies have evidenced the beneficial effects of community group singing on various aspects of mental health [10, 16, 17]. Singing to new babies is a common practice utilised cross-culturally around the world, and studies show that singing to newborns not only improves maternal mood, aspects of self-esteem, and sense of wellbeing, but also enhances the developing bond with a baby, aids in the mother-infant interaction, and helps to soothe babies in distress [10, 18–21]. Taken together, these studies suggest that singing is an efficacious way to support new mothers who are struggling with depressive symptoms and their babies.

Breathe Melodies for Mums (M4M) is a singing intervention that was developed by the Breathe Arts Health Research on the basis of research evidence generated as part of a collaboration between the Royal College of Music, Imperial College London, and University College London from 2015 to 2017. The programme involved weekly singing classes for mothers and their babies delivered in Children’s Centres for 10 weeks. The intervention, which was tested in a three-arm RCT involving 134 mothers with PND, found that mothers with moderate to severe symptoms of PND (EPDS ≥ 13) who took part in the singing programme with their baby had a significantly faster improvement in symptoms than mothers who were in the usual care arms of the study [22]. Specifically, it was found that mothers in the singing group had an overall EPDS scores reduction by 35% during the first 6 weeks of intervention and, furthermore, that after just 6 weeks, 65% of the singing mothers already no longer had an EPDS score indicating more than mild depression.

Possible mechanisms underlying these changes included an increase in the frequency of mothers singing to their babies outside of the classes, improvements in their confidence in singing, and increases in the breadth of their singing repertoire [23]. Moreover, group singing improved perceived mother-infant closeness and led to a greater decrease in the stress hormone, cortisol, than social play [24].

#### M4M during COVID-19

While we have funding to upscale M4M as part of the SHAPER-PND programme [25, 26] (a Hybrid Type II Effectiveness-Implementation randomised-control trial investigating the clinical and implementation effectiveness of the face-to-face singing intervention on symptoms of PND) [27, 28] funded by the Wellcome Trust, the recent lockdown has so far not only halted the delivery of the programme in its face-to-face format, but also prompted the necessity to develop an online version of the intervention that can be used: (1) if the requirement for social distancing continues, even when the lockdown is relaxed, making the delivery of the programme difficult; and (2) to broaden our reach to a nationwide delivery and extend to a wider population who may not have otherwise been able to attend in-person sessions due to treatment barriers including geographical, physical, or financial constraints, because of the severity of their depressive or anxious symptoms, and/or because of healthcare or policy barriers preventing adequate treatment pathways. In order to address the specific treatment barriers listed in the ‘Barriers to treatment’ section above, we will recruit participants across multiple platforms that are not necessarily mental health-related (i.e. through social media, word-of-mouth, and at baby centres), engage multiple types of healthcare practitioners to inform them of the study and the criteria for postnatal depressive symptoms, and deliver the intervention on an online platform that has much greater reach than other care options.

While community group singing for PND has typically involved face-to-face sessions, results of online interventions, such as cognitive behavioural therapy, for mental health problems including anxiety disorders and depression have been encouraging [29]. Additionally, such treatments appear to have similar efficacy to face-to-face therapy [30]. Online interventions specifically for mothers with PND offer promising results in overcoming some of the main barriers to treatment access [20], as they reduce obstacles such as perceived stigma, fear of contracting COVID-19 from social contacts, they are more easily accessible, and can target groups of women who would struggle to attend in-person sessions. Thus, online treatments hold the potential to empower women with depression to take effective and manageable steps to overcome their mental health difficulties. However, there is currently a lack of research examining the effectiveness and feasibility of an online singing intervention for mothers with PND, including whether it may present with its own unique set of challenges, e.g. digital poverty; thus, further research is warranted.

In support of our approach, research on the emotional impact of online (or ‘virtual’) singing as part of a choir has shown a high degree of personal engagement, even when using quite primitive ‘virtual’ settings. For example, a study by Fancourt and Steptoe (2019) has shown that attending a ‘virtual choir’, where participants recorded their performances in their own individual physical localities and then the performance was combined and presented back in cyberspace, can lead to equivalent feelings of the social presence as participating in live choirs would. Participants felt a ‘sense of connection’ to the other people who were doing the same activity and felt part of ‘a community’ and of ‘something bigger’ [31], even if they had not met in person.

### Aims

This single-arm feasibility study aims, in a period of 12 months, to evaluate an online delivery of Melodies for Mums with the ambition to develop a remote intervention that can become a mainstream therapeutic tool not only in times of social isolation and distancing, but also for mothers who cannot leave their houses (e.g. because of mental health difficulties, medical comorbidities, or financial difficulties) or who live in areas where the face-to-face intervention is unavailable.

### Objectives

#### Primary feasibility objectives

The primary objective of this study is to assess the feasibility and acceptability of a group online singing intervention for new mothers with postnatal depression in order to ensure adequate recruitment for a future RCT. We will thus assess recruitment rates, retention rates, study completion rates, acceptability of intervention, and scalability to power a subsequent RCT.

#### Patient-centred objectives

In order to assess the clinical efficacy of our study, we have the following patient-centred objectives:

Primary

- To assess the effectiveness of online singing for symptoms of postnatal depression

Secondary

- To assess whether online singing improves further aspects of mental health, including anxiety and stress
- To ascertain whether online singing affects the mother-infant relationship
- To ascertain whether online singing improves perceived social support and reduces loneliness
- To identify whether there are biological changes in stress mechanisms underpinning the psychological outcomes assessed
- To explore the uptake and continued involvement in online singing groups

## Methods

### Trial design

In light of the limitations imposed by the current pandemic, we aim to deliver the M4M programme using a virtual platform to replace the in-person singing sessions. To achieve this, we will deliver a more advanced version of a ‘virtual choir’ while keeping to the framework of the existing M4M programme; we will take the learnings by the Breathe Arts Health Research from their Breathe Sing group for individuals with respiratory conditions. Prior to lockdown, this group met fortnightly in-person to use singing to benefit physical and mental health. During the COVID-19 pandemic, this group has shifted online and is continuing to have excellent uptake with weekly attendance numbers in fact higher than when the group was delivered in-person (November 2019 attendance of 8 women, compared with November 2020 attendance of 13 women).

M4M online will take a similar format, consisting of weekly sessions of 1 h each, during which women will connect via Zoom at the designated date and time, and sing from their home while following the singing leader. To avoid the inevitable problems with Wi-Fi delays and instability, all participants are muted at certain times in the session but can all hear the singing leader throughout. The singing leader will also use a backing track that will be recorded specifically to support online singing delivery and has other voices and harmonies included to amplify the experience of singing with others and to try new singing parts. An additional Breathe member of staff will support participants with setting up the online connection before the session starts and help them with troubleshooting during the session. All participants will also be connected via a WhatsApp group (monitored by Breathe) on which they can communicate outside of class, and finally, there will be time at the beginning and end of the sessions where all participants are unmuted and can chat with the artist or their peers, in an unstructured way.

M4M online will be delivered as a single-arm clinical trial. Participants will be recruited in the community, mostly via social media and other signposting methods according to the usual procedures carried out by Breathe for recruiting into the face-to-face M4M programme but with enhanced advertisement and social media reach nationally. Before entering the study, mothers will be assessed by the research team, and if found eligible, they will be allocated to a singing group for 6 weekly sessions (M4M online is a 6-week programme rather than a 10-week programme, as in the face-to-face study already by week 6, we found a significant improvement in depressive symptoms compared with control interventions). As it is a single-arm trial, neither participants nor researchers will be blinded to study condition. Participants will remain in the group allocated to them to allow for a greater sense of community and familiarity with the artist, the facilitator, and the other participants. Participants will be regularly assessed by the researchers and if they give consent, they will provide biological samples. Follow-ups will be carried out at 16 and 32 weeks, that is, 10 and 26 weeks after completion of the singing sessions.

The SHAPER-PNDO single-arm trial obtained ethical approval from the King’s College London Research Ethics Office on 25 November 2020 (KCL Ethics Ref: HR-20/21–19,659) and has been registered with ClinicalTrials.gov, trial number NCT04857593.

### Study participants and sample size rationale

The number of participants to recruit to this feasibility intervention is defined by the maximum number of mothers that Breathe can place in an online singing session and the number of singing groups that logistically can be delivered in 12 months. There will be 4 groups in total of 30 participants each (split into two Zoom sessions), totalling 120 participants recruited for the study.

Participants (mothers) will be recruited in blocks of 40–50 women and screened and assessed prior to enrolment. Each block of singing sessions will aim to have around 30 women divided into two sessions. This process will be repeated 4 times, aiming to reach 120 participants in total. Furthermore, amongst the 120 women recruited to the intervention, we will further recruit 20–30 to take part in subsequent qualitative interviews, in addition to the intervention deliverers.

#### Study population

Study participants will be new mothers with symptoms of PND.

#### Eligibility criteria

Women will be eligible for the study if they:

- Are aged 18 or older
- Have a satisfactory understanding of English
- Have a child between 0 and 9 months old
- Have symptoms of PND, defined as a minimum score of 10 on the EPDS

#### Exclusion criteria

Women will not be able to enter the study if ANY of the following apply:

- Child outside of the age range specified
- Unable to give informed consent
- Unable to access online sessions (i.e. Internet connection, laptop, or computer availability)

### Trial setting

This is a single-centre trial that will be run online across the UK via the platform Zoom. In order to enrol a sufficiently large sample of women, there will be 4 blocks of the 6-week singing sessions in total. Women will be followed up at weeks 3, 6, 16, and 32 after baseline. 

### Study flowchart

**Fig. 1:**
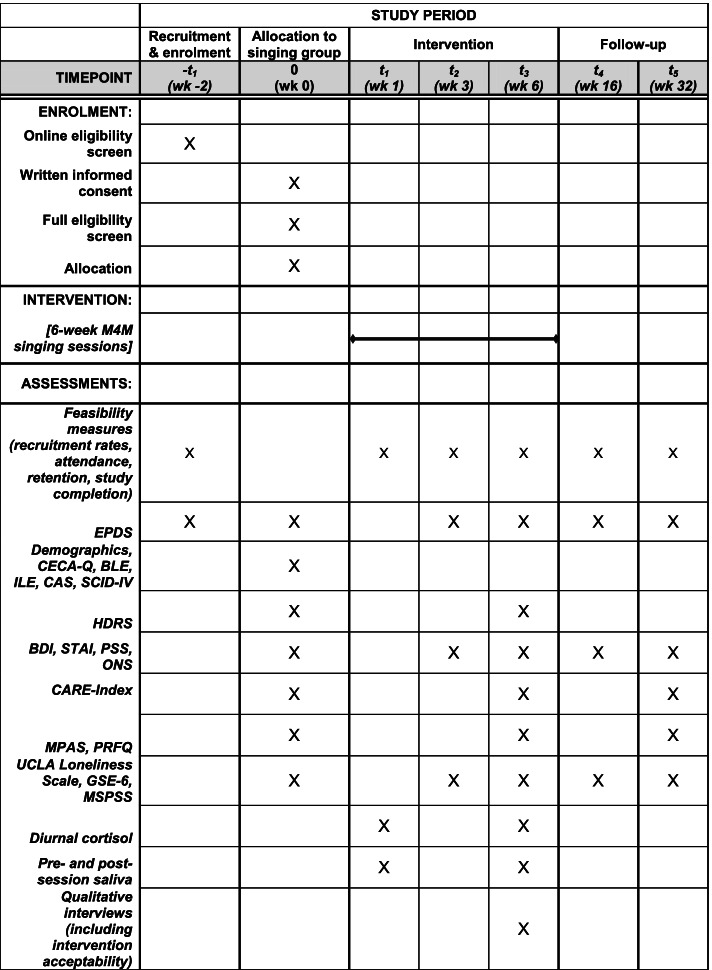
Schedule of enrolment, interventions, and assessments

This will be a non-randomised, single group trial using the following timeline procedure presented in Fig. 1.

### Intervention

M4M online is a 6-week intervention for mothers with PND (and their babies), adapted from the original M4M programme [10], which would be delivered face-to-face in groups of 8–12 mothers in weekly sessions lasting 1 h; however, due to the current pandemic and government restrictions, we will modify the original face-to-face intervention for this online study, as follows:

- We will run groups of around 15 women to ensure that all participants can be visible on one screen during online delivery to create a stronger sense of community and connection.
- We will offer 6 weeks of intervention, building on the evidence from the face-to-face intervention that by 6 weeks there is already a significant improvement in depressive symptoms compared with control interventions [22].
- We will connect mothers during week 2 of the intervention to an optional WhatsApp group so they can get to know each other.

As in the face-to-face M4M intervention [10], classes will start with a chat between mothers and the artist before the start of the singing session. The singing session will comprise welcome songs, introducing the babies and mothers to one another, and then a range of singing and music activities. These will include learning songs from around the world, ranging from short vocal exercises that use ‘motherese’ style noises and sound effects (including sound baths where the mothers sing a sustained note providing a relaxation technique), to simple lullabies that can be picked up very quickly and sung in basic harmonies or rounds, and to longer or more complex songs that will be learnt gradually over the weeks. Songs will range in style from relaxing, with mothers encouraged to hug or stroke their babies as they sing, to energetic, with mothers standing and moving with their babies and bouncing their babies in their arms. Instruments such as guitars and ukuleles will also be used by the artist for a small number of songs. Mothers will also work to write some of their own songs over the weeks, developing lyrics together about their babies or experiences of motherhood and creating simple melodies. Recordings of the group singing the songs together will be made and uploaded to private online platforms for the mothers to listen to at home. Classes will be led by professional workshop leaders trained by Breathe, with the support of assistants.

### Study procedures

#### Recruitment and informed consent

##### Participants

Participant recruitment will be primarily through posters and flyers in baby weigh clinics and other community and clinical centres for postnatal mothers and their babies, if lockdown rules allow for it. Additionally, we will signpost via other health and social care professionals and in the community via email contacts, advertisement on social media, and online forums. We will also accept participants via self-referral following general advertisement.

The recruitment period will last approximately 1 year. All potential participants will initially be directed to a pre-screening online form that includes the EPDS. If the EPDS total score is < 10, the participants will be notified that they are not currently eligible to participate and will be signposted to other support services within the community (e.g. talking therapies, mother-baby groups, baby activity groups). If the score is ≥ 10, the participants will be notified that they are potentially eligible and will proceed straight to the second phase of screening through a Zoom meeting. The participant information sheet (PIS) and informed consent form (ICF) for the trial will be sent once the baseline assessment is booked.

**Fig. 2:**
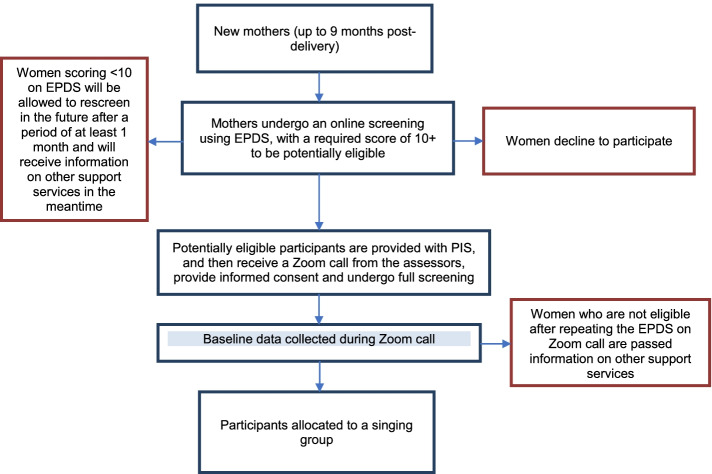
Recruitment strategy

When a new round of 6 classes becomes available, researchers will arrange assessments with potential participants to undertake the full screening interview against the inclusion/exclusion criteria. If the participant is found not eligible to take part, she will be signposted to other support services within the community. Our recruitment strategy is presented as a flow chart in Fig. 2.

##### Deliverers

During the course of the study, deliverers will be recruited by the research team from the pool of 1–3 artists trained by Breathe that deliver the M4M online sessions. We will approach deliverers of the intervention to ascertain their interest level in participating in our research study. Deliverers will be recruited to the study for the purpose of assessing the ways in which the 6-week online singing programme can ameliorate mothers’ postnatal depression.

If deliverers express interest, we will provide them with the Deliverer PIS and ICF, which will explain to them in better detail why we are interested in incorporating their feedback on the programme into our study. If deliverers consent to participation, a researcher will arrange an interview with them to explore the barriers and facilitators to delivering the online singing groups and how they felt the programme helped women’s postnatal depression. This will help inform upon further development and modifications to the online programme.

### Safeguarding and participant care

No significant risks to physical safety are expected; however, changes in depressive symptoms may occur in mothers during the trial and cause psychological harm. Although a formal data monitoring committee is not required due to the minimal physical risks associated with the intervention, in order to ensure participant safety, the research team will check the EPDS scores of all participants as they complete questionnaires throughout the study. Safeguarding leads will be alerted if EPDS scores are above 25, or if participants report self-harm thoughts ‘sometimes’ or ‘quite often’. Additionally, deliverers of the music sessions or researchers will alert safeguarding leads if any concerning behaviour is observed during sessions or assessments. If the safeguarding lead deems it appropriate, a participant’s GP or other healthcare provider will be contacted to ensure appropriate support is provided. Any related or unexpected serious adverse events will be reported to the Research Ethics Committee.

#### Feasibility assessments

To assess the feasibility of our online intervention, we will track recruitment rates for each block of sessions and total recruitment across all blocks, attendance rates for each session and overall attendance across each block, the population demographics that our study reaches, our participant retention throughout the intervention period as well as through the follow-up period, and our study completion rates both during the intervention period as well as through the follow-up.

Furthermore, individual qualitative interviews will be conducted with a sub-sample of participants after the final intervention session, either by telephone or video call. In addition, interviews will also take place with the intervention deliverers/singing group leaders. The interviews will explore the acceptance and acceptability of the intervention, lived experiences, and barriers and facilitators of taking part, in order to prepare for our subsequent RCT. Interviews will be conducted until no new themes or concepts are identified within the data.

### Patient-centred assessments

Participants will undergo clinical assessments via Zoom at baseline, week 6, and week 32. Week 3 and 16 assessments will be completed fully online by the mothers, due to the self-reporting nature of the measures to be captured. Additionally, mothers will have online questionnaires to fill out at baseline, week 6, and week 32 that do not need to be completed over Zoom. It is expected that all mothers will have access to an Internet-enabled device, as this is an inclusion criterion. For baseline online measures, participants will be encouraged to complete these a day before or after the first session. For week 3 measures, participants will be encouraged to complete these within 3 days of the session. However, in order to allow for flexibility in the schedule, a ± 1-week variation in the date of collection of the measures below will be accepted (apart from week 6, when the window will be weeks 6–8).

Individual qualitative interviews with a sub-sample of women will take place after the final intervention session and 6-week assessment, either by telephone or video call. Interviews will also be conducted with the intervention deliverers/singing group leaders. These interviews will explore the clinical mechanisms of effect of taking part and delivering online singing groups. Interviews will be conducted until no new themes or concepts are identified within the data.

Table 1 presents our data collection plan and timeline for both our feasibility and patient-centred objectives.

**Table 1:** Data collection plan

Type of measure	Measures	Timepoint collected	Type of data
Feasibility	Recruitment, attendance, retention and withdrawal rates, study completion	Throughout intervention period	Feasibility
	Semi-structured interviews with a sub sample of 20–30 women and intervention deliverers to understand intervention uptake, acceptance, and acceptability	Week 6	
Mental health: screening tool for PND	Edinburgh Postnatal Depression Scale (EPDS) (33)	Baseline, weeks 3, 6, 16, 32	Quantitative
Demographic information	Demographics interview, Childhood Experience of Care and Abuse-Questionnaire (CECA-Q) (34), Brief Life Events Scale (35), Intrusive Life Events Scale, Composite Abuse Scale (CAS) (36)	Baseline	
Mental health: depression, anxiety, wellbeing, stress	Structured Clinical Interview for DSM-IV Disorders (SCID-IV) (37)	Baseline	
	Hamilton Depression Rating Scale (HDRS) (38)	Baseline, week 6	
	Beck Depression Inventory (BDI) (39), State Trait Anxiety Inventory (STAI) (40), Perceived Stress Scale (PSS) (41), Office for National Statistics Wellbeing Scale (ONS) (42)	Baseline, weeks 3, 6, 16, 32	
Social: mother-infant relationship	Maternal Postpartum Attachment Scale (MPAS) (43), Parental Reflective Functioning Questionnaire (PRFQ) (44)	Baseline, weeks 3, 6, 16, 32	
	Crittenden CARE-Index (CCI) (45)	Baseline, weeks 6, 32	
Social: loneliness and social support	UCLA Loneliness Scale (46), Short General Self-Efficacy Scale (GSE-6) (47), Multidimensional Scale of Perceived Social Support (MSPSS) (48)	Baseline, weeks 3, 6, 16, 32	
Biological markers	Diurnal cortisol saliva samples	Baseline, week 6	
	Pre- and post-session saliva samples	Singing sessions 1, 6	
Qualitative interview	Semi-structured interviews with a sub sample of 20–30 women and intervention deliverers for clinical mechanisms of effect	After week 6	Qualitative

#### Biological samples

All saliva samples will be collected by Salivette absorbent swabs for adults and SalivaBio Children’s Swabs for babies and will be used to measure cortisol levels. Mothers will be asked to collect samples to measure diurnal cortisol rhythm and cortisol reactivity to the sessions.

For diurnal samples, mothers will be asked to collect six saliva samples from themselves (awakening, + 15, + 30, and + 60 min after awakening, at 12 noon and 8 pm) and two samples from their baby (awakening and 8 pm). Mothers will be asked to collect these samples up to 3 days prior to their session (baseline and week 6); however, in order to allow for flexibility in the schedule, a ± 5 day variation from session date will be accepted in the date of collection. We have extensive experience with this methodology and have successfully collected such data before [32].

Mothers will also be asked to collect saliva samples from themselves and their baby immediately before and after their session (session 1 and session 6).

#### Follow-up data collection

Upon termination of the 6 weekly sessions, participants will be contacted to complete follow-up questionnaires around weeks 16 and 32. An additional virtual assessment will be carried out around week 32. A sub-sample of women will be invited to take part in a qualitative interview about their experiences of receiving the online intervention up to 2 weeks after their final intervention session. In order to compensate participants for their time, and improve participant retention, we will provide participants with £20 high street vouchers per timepoint assessment. If participants choose to withdraw from the study, no further data will be collected.

### Outcomes

The outcomes for both our feasibility and patient-centred study objectives are presented in Table 2.

**Table 2:** Study objectives and outcomes

**Primary feasibility objectives**	**Outcome measures/endpoints**
To assess the feasibility and acceptability of a group online singing intervention in order to ensure adequate recruitment for a future RCT	We will assess recruitment rates, retention rates, study completion rates, acceptability of intervention, and scalability to power a subsequent RCT
**Secondary patient-centred objectives**	**Outcome measures/endpoints**
To assess the effectiveness of a group online singing intervention on symptoms of postnatal depression	Depressive symptoms measured before, during, and after the intervention using the EPDS The secondary outcome measure is change in total EPDS score between baseline and week 6 (endpoint of intervention)
To assess whether online singing improves further aspects of mental health	Depressive, anxious, and stress symptoms measured before, during, and after the intervention using the SCID, HDRS, BDI, ONS, STAI, and PSS The secondary outcome measure is change in total scores between baseline and week 6 (endpoint of intervention)
To ascertain whether online singing affects the mother-infant relationship	Aspects of the mother-infant relationship measured before, during, and after the intervention using the CCI, MPAS, and PRFQ The secondary outcome measure is change in total scores between baseline and week 6 (endpoint of intervention)
To ascertain whether online singing improves perceived social support and reduces loneliness	Social support and loneliness measured before, during, and after the intervention using the UCLA Loneliness Scale, MSPSS, and GSE-6 The secondary outcome measure is change in total scores between baseline and week 6 (endpoint of intervention)
To identify whether there are biological changes in stress mechanisms underpinning the psychological outcomes assessed	Cortisol from saliva samples The secondary outcome measure is change in circulating biomarkers
To explore the uptake and continued involvement in online singing groups	Qualitative interviews with a sub-sample of 20–30 women and the intervention deliverers

### Statistical methods

#### Quantitative analyses

Analyses for our primary feasibility objectives will be reported as descriptive statistics with 95% confidence intervals listed.

Analyses for our patient-centred objectives will be hypothesis-driven, and we will test within-subject changes in the clinical outcome data (EPDS) using repeated-measures analysis of covariance. The within-person association between the primary outcome (changes in the EPDS total score) and secondary outcomes (for example, changes in other psychiatric symptoms, in mother-infant interaction, or in cortisol secretion) will be analysed using fixed effects regressions with data from 5 timepoints (baseline, 3, 6, 16, and 32 weeks post baseline). Missing data will be dealt with by multiple imputation (MI) under the missing at random (MAR) assumption. Departures from this assumption will be assessed with a sensitivity analysis using only available data. Mediation analysis with the use of structural equation models will also be employed to understand the potential pathways in which changes in the secondary outcomes have an impact on the effectiveness of M4M. Findings will be reported with both *p* values and 95% confidence intervals and will be considered exploratory. All analyses will be conducted using STATA V.15.1.

#### Qualitative analyses

Qualitative interviews with mothers receiving the online intervention will capture general feedback from sessions and experiences from mothers in the groups, focusing on the lived experience of PND and how this intersects with experiences in the singing group. We will explore, with both mothers receiving the intervention and intervention deliverers, their experiences of receiving and delivering the intervention online, mechanisms of effect, barriers, and facilitators to taking part in the intervention and in their continued involvement.

All interviews will be audio-recorded and transcribed by a UCL approved external transcription company. Transcripts will be anonymised before analysis. Interviews will be analysed using thematic analysis [33]. All analysis will be conducted using NVivo 12. Coding and organisation of codes will be cross-checked within the research team to ensure validity.

## Discussion

To our knowledge, the SHAPER-PNDO study is the first of its kind to investigate the feasibility of an online singing intervention for new mothers with postnatal depression, particularly during times of social isolation, especially relevant during the COVID-19 pandemic. By offering a remote intervention to mothers and their babies all over the UK, we hope to develop a new and effective therapeutic option for PND that can become a mainstay not only during a pandemic, but also beyond this timepoint. Given the complexities and barriers with which women with depression can present and face, including not being able to leave their homes or access treatment, online interventions are an especially important treatment pathway to consider going forward. Should the M4M online singing trial prove effective, it will pave the way for a new and accessible type of intervention for mothers and their babies across the UK. Furthermore, by refining our recruitment process, data collection procedures, and running of the intervention itself, from this feasibility trial, we aim to prepare for a much larger subsequent hybrid RCT in which we will compare outcomes between our Melodies for Mums intervention (both online and in-person) with care as usual.

## Supplementary Information

**Additional file 1.** Saliva sample collection, storage and analysis procedures.

**Additional file 2.** Saliva collection booklet

## Data Availability

Not applicable, the manuscript does not contain any data.

## Abbreviations

BDI: Beck Depression Inventory
CCI: Crittenden Child–Adult Relationship Experimental (CARE)-Index
CECA-Q: Child Experience of Care and Abuse-Questionnaire
CI: Chief investigator
DOB: Date of birth
eCRF: Electronic case report form
EPDS: Edinburgh Postnatal Depression Scale
GP: General practitioner
GSE-6: Short General Self-Efficacy Scale
HDRS: Hamilton Depression Rating Scale
ICF: Informed consent form
KCL: King’s College London
M4M: Melodies for Mums
MPAS: Maternal Postnatal Attachment Scale
MSPSS: Multidimensional Scale of Perceived Social Support
ONS: Office for National Statistics
PIS: Participant/patient information sheet
PND: Postnatal depression
PSS: Perceived Stress Scale
REC: Research Ethics Committee
SCID: Structured Clinical Interview for DSM-IV Disorders
STAI: State Trait Anxiety Inventory
UCL: University College London
