# Supplementary material for: Online singing interventions for postnatal depression in times of social isolation: a feasibility study protocol for the SHAPER-PNDO single-arm trial
Source: Pilot Feasibility Stud. 2022 Jul 18;8:148. doi: 10.1186/s40814-022-01112-1 (PMC9289358; doi:10.1186/s40814-022-01112-1)
Supplement: Supplementary file 1 — Additional file 1. Saliva sample collection, storage and analysis procedures. [file 40814_2022_1112_MOESM1_ESM.pdf]

**APPENDIX: Saliva Sample Collection, Storage and Analysis Procedures**

**Collection [taken from manuscript]**

All saliva samples will be collected by Salivette absorbent swabs for adults and SalivaBio Children's Swabs for babies and will be used to measure cortisol levels. Mothers will be asked to collect samples to measure diurnal cortisol rhythm and cortisol reactivity to the sessions.

For diurnal samples, mothers will be asked to collect six saliva samples from themselves (awakening, +15, +30, and +60 min after awakening, at 12 noon and 8 pm), and two samples from their baby (awakening and 8 pm). Mothers will be asked to collect these samples up to 3 days prior to their session (baseline and week 6); however, in order to allow for flexibility in the schedule, a +/-5 day variation from session date will be accepted in the date of collection. Mothers will also be asked to collect saliva samples from themselves and their baby immediately before and after their session (session 1 and session 6).

**Transport and Storage**

Participants will be instructed to keep their saliva samples in the fridge until all have been collected. They will then return their samples to King's College London via mail, in UN3373 category B-compliant packaging, provided by researchers. Samples will be stored at -20$^{o}$C at the Maurice Wohl Clinical Neuroscience Institute (King's College London) pending analysis.

**Sample Analysis**

Cortisol concentration in saliva samples will be quantified using a commercially available ELISA kit (Salimetrics Europe Ltd, UK), according to manufacturer's protocols.
