# Supplementary material for: Online singing interventions for postnatal depression in times of social isolation: a feasibility study protocol for the SHAPER-PNDO single-arm trial
Source: Pilot Feasibility Stud. 2022 Jul 18;8:148. doi: 10.1186/s40814-022-01112-1 (PMC9289358; doi:10.1186/s40814-022-01112-1)
Supplement: Supplementary file 2 — Additional file 2. Saliva collection booklet [file 40814_2022_1112_MOESM2_ESM.docx]

| **Participant ID** |  | - |  |  |  |  |  |  |  |  |
| --- | --- | --- | --- | --- | --- | --- | --- | --- | --- | --- |
|  |  |  |  |  |  |  |  |  |  |  |
| **Participant DOB** | *d* | *d* | *-* | *m* | *m* | *-* | *y* | *y* | *y* | *y* |
|  |  |  |  |  |  |  |  |  |  |  |
| **Baby DOB** | *d* | *d* | *-* | *m* | *m* | *-* | *y* | *y* | *y* | *y* |
|  |  |  |  |  |  |  |  |  |  |  |
| **Researcher’s Initials** |  |  |  |  |  |  |  |  |  |  |

**SALIVA COLLECTION BOOKLET**

Week 0 Week 3 Week 6 **(please circle as appropriate)**

**
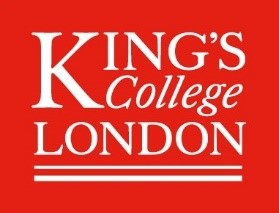
**

**INSTRUCTIONS FOR SALIVA COLLECTION**

Thank you very much for your willingness to collect saliva samples. There are 3 sets of samples for you to collect.

SET 1: Mother daily samples

Six samples throughout the course of a day (4 within first hour of waking up)

SET 2: Baby daily samples

Two samples in a day (wakening and evening)

SET 3: Session day samples (from both mother and baby before and after singing session)

Please note:

- All samples from set 1 should be collected on the **same day**
- Both set 2 samples should be collected on the **same day**
- Sets 1 and 2 do not need to be collected on the same day as each other
- Sets 1 and 2 should be collected **no more than 3 days prior** to your first singing session.

All tubes are labelled. Please make sure you select the correct tube for each sample you collect.

In the next few pages you will find more details on each of the sets, and space for you to complete information on the day of collecting the samples. Please make this information as accurate and complete as you can.

At the end of the booklet there are instructions for how to collect the samples, and how to post your samples back to us. Please note, once collected, saliva samples should be kept in your fridge if possible until you can post them all back to us in the packaging provided.

*****Please note: if you or anyone you have been in contact with has symptoms or tests positive for coronavirus, please DO NOT collect saliva samples, and inform the research team by emailing shaperpndo@kcl.ac.uk. Please also contact the research team if you or anyone in your household develops symptoms up to 2 weeks after returning samples to us.*****

**If you have any questions at all, please email the team on shaperpndo@kcl.ac.uk**

**SET 1: MOTHER DAILY sampleS (6 samples)**

|  |  |  |  |  |  |  |  |  |  |  |  |  |  |
| --- | --- | --- | --- | --- | --- | --- | --- | --- | --- | --- | --- | --- | --- |
| **dATE of collection (DD-MM-YY)** | | | | | |  |  | - |  |  | - |  |  |

**IMPORTANT NOTES:**

- Please collect the first sample of saliva **immediately after waking up**. You may find it helpful to keep the first tube next to your bed.
- Please **do not** eat, drink, smoke or brush your teeth between samples at 0, 15, 30 and 60 minutes.
- Please **do not** eat, drink, smoke or brush your teeth 30 minutes before samples at Midday and 8 pm.
- You may drink water if you need to, but only immediately **after** you have taken a sample.
- Collect the saliva as on the instruction diagram. Place the swab in your mouth and leave it there for 1-2 minutes, then place it as shown in the correct tube. Then close the tube firmly and store in the fridge in the bag supplied.
- Try to sit down and relax for the first hour after waking up.

| **Please record the EXACT TIME you woke up:** | | **_________________** |
| --- | --- | --- |
| **Complete** | **When?** | **Record the time of saliva collection:** |
| BOX 1 (Tube MOTHER 0) | When you wake up (Before 10am) |  |
| BOX 2 (Tube MOTHER 15) | 15 minutes after you wake up |  |
| BOX 3 (Tube MOTHER 30) | 30 minutes after you wake up |  |
| BOX 4 (Tube MOTHER 60) | 60 minutes after you wake up |  |
| BOX 5 (Tube MOTHER 12pm) | Midday (12:00) |  |
| BOX 6 (Tube MOTHER 8pm) | 8 o’clock pm (20:00) |  |

| **Sample 1:** When you wake up (before 10am) **USE TUBE MOTHER 0** |
| --- |
| - What time is it now?________________________________________________________ - Did you accidentally brush your teeth, smoke or have anything to eat or drink before taking the sample? If yes, please describe it here and record the time: ________________________________________________________________________ - Did you have any difficult or tense situation, unpleasant thoughts or any kind of pain before taking this sample? If yes, please describe it here: __________________________________________________________________________________________________________________________________________________ |

| **Sample 2:**  15 minutes after waking up **USE TUBE MOTHER 15** |
| --- |
| - What time is it now? ______________________________________________________ - What were you doing before giving the sample? ______________________________________________________________________ - Did you accidentally brush your teeth, smoke or have anything to eat or drink before taking the sample? If yes, please describe it here and record the time: ______________________________________________________________________ - Did you have any difficult or tense situation, unpleasant thoughts or any kind of pain before taking this sample? If yes, please describe it here: __________________________________________________________________________________________________________________________________________________ |

| **Sample 3:** 30 minutes after waking up **USE TUBE MOTHER 30** |
| --- |
| - What time is it now? ________________________________________________________ - What were you doing before giving the sample? ________________________________________________________________________ - Did you accidentally brush your teeth, smoke or have anything to eat or drink before taking the sample? If yes, please describe it here and record the time: ________________________________________________________________________ - Did you have any difficult or tense situation, unpleasant thoughts or any kind of pain before taking this sample? If yes, please describe it here: ________________________________________________________________________   ________________________________________________________________________ |

| **Sample 4:** 60 minutes after waking up **USE TUBE MOTHER 60** |
| --- |
| - What time is it now? ________________________________________________________ - What were you doing before giving the sample? ________________________________________________________________________ - Did you accidentally brush your teeth, smoke or have anything to eat or drink before taking the sample? If yes, please describe it here and record the time: ________________________________________________________________________ - Did you have any difficult or tense situation, unpleasant thoughts or any kind of pain before taking this sample? If yes, please describe it here: __________________________________________________________________________________________________________________________________________________ |

*******YOU CAN NOW EAT, DRINK, SMOKE AND BRUSH YOUR TEETH! *******

Please **do not eat, drink, smoke or brush your teeth** for **30 minutes** before collecting the **sample at Midday**

| **Sample 5:** Midday (12:00pm) before lunch **USE TUBE MOTHER 12pm** |
| --- |
| - What time is it now? _______________________________________________________ - What were you doing before giving the sample? ________________________________________________________________________ - Did you accidentally brush your teeth, smoke or have anything to eat or drink before taking the sample? If yes, please describe it here and record the time: ________________________________________________________________________ - Did you have any difficult or tense situation, unpleasant thoughts or any kind of pain before taking this sample? If yes, please describe it here: _______________________________________________________________________   ________________________________________________________________________ |

*******YOU CAN NOW EAT, DRINK, SMOKE AND BRUSH YOUR TEETH AGAIN! *******

Please **do not eat, drink, smoke or brush your teeth** for **30 minutes** before collecting the **sample at 8 o’clock pm**

| **Sample 6:** At 8 o’clock pm (20:00pm) **USE TUBE MOTHER 8pm** |
| --- |
| - What time is it now? __________________________________________________________ - What were you doing before giving the sample?   _______________________________________________________________________   - Did you accidentally brush your teeth, smoke or have anything to eat or drink before taking the sample? If yes, please describe it here and record the time: __________________________________________________________________________ - Did you have any difficult or tense situation, unpleasant thoughts or any kind of pain before taking this sample? If yes, please describe it here:   __________________________________________________________________________  _____________________________________________________________________________ |

Please note the name and time of **any medication you have taken today** (including the contraceptive pill): _____________________________________________________________________________

Have you had any vaccinations in the past two weeks? If so, please provide details and date:

_____________________________________________________________________________

Do you have any medical problems? If so, please list them here: _____________________________________________________________________________

**Please remember to place all your samples in the fridge until posting.**

**SET 2: BABY DAILY SAMPLES (2 samples)**

|  |  |  |  |  |  |  |  |  |  |  |  |  |  |
| --- | --- | --- | --- | --- | --- | --- | --- | --- | --- | --- | --- | --- | --- |
| **dATE of collection (DD-MM-YY)** | | | | | |  |  | - |  |  | - |  |  |

**N.B. Ideally the samples should not be taken for at least 15 minutes after a feed.**

| **Immediately after your baby wakes up and before feeding** collect your baby’s saliva using the swab as shown on the instruction diagram. Place the swab ***under*** your ***baby’s tongue*** and leave them there for a total of 60-90 seconds (the swab can be placed under the tongue for 15 to 30 seconds at a time and reintroduced as needed), then place them back in the **tube LABELLED “BABY WAKE”**, close the tube firmly and store in the bag supplied.  **EXACT TIME OF SAMPLING:** ________________________  What time did your baby wake up? _______  What time was your baby’s most recent feed? Start: ________ Finish: ________ |
| --- |

| **At 8pm** collect your baby’s saliva using the swab and place in the **tube labelled “BABY 8 PM”.**  Then close the tube firmly and **store in the fridge** in the bag supplied  **EXACT TIME OF SAMPLING:** ________________________  What time was the baby’s most recent nap? Start: ________ Finish: ________  What time was the baby’s most recent feed? Start: ________ Finish: ________ |
| --- |

Please note the name and time of any medication your baby has taken today: _____________________________________________________________________________

Has your baby had any vaccinations in the past two weeks? If so, please provide date and details:

_____________________________________________________________________________

Does your baby have any medical problems? If so, please list them here: _____________________________________________________________________________

**SET 3: SINGING SESSION DAY SAMPLES (4 SAMPLES)**

| **dATE of SESSION (DD-MM-YY)** |  |  | - |  |  | - |  |  |
| --- | --- | --- | --- | --- | --- | --- | --- | --- |

Please collect samples from yourself and your baby immediately prior to the singing session, and as soon as possible after the session has finished. You should collect these in the same way as you did for sets 1 and 2.

Please try not to eat/drink/smoke/brush teeth for 30 minutes prior to taking the samples. Ideally baby would not be fed for 15 minutes prior to taking samples.

| **Please record the START TIME of the session:** | | **_________________** |
| --- | --- | --- |
| **Complete** | **When?** | **Record the EXACT time of saliva collection:** |
| Mother pre-session sample  (Tube MOTHER PRE) | Immediately **before** session starts |  |
| Baby pre-session sample  (Tube BABY PRE) |  |  |
| **Please record the END TIME of the session:** | | **_________________** |
| Mother post-session sample (Tube MOTHER POST) | Immediately **after** session ends |  |
| Baby post-session sample (Tube BABY POST) |  |  |

**Comments e.g. Was baby distressed during? How did you feel during session?**

**Please turn over to complete information about session day.**

**Mother Sample Information**

Did you accidentally brush your teeth, smoke or have anything to eat or drink before taking the sample? If yes, please describe it here and record the time: ________________________________________________________________________

Please note the name and time of **any medication you have taken today** (including the contraceptive pill): _________________________________________________________________________

Have you had any vaccinations in the past two weeks? If so, please provide date and details:

_________________________________________________________________________

Do you have any medical problems? If so, please list them here: _________________________________________________________________________

**Baby Sample Information**

What time was baby’s most recent nap? Start: ________ Finish: ________

What time was baby’s most recent feed? Start: ________ Finish: ________

Please note the name and time of any medication your baby is taking

_________________________________________________________________________

Has your baby had any vaccinations in the past two weeks? If so, please provide date and details

_________________________________________________________________________

Does your baby have any medical problems? If so, please list them here:

**RETURNING YOUR SAMPLES TO US**

Once collected, please place sample tubes back into the clear plastic bag they came in and store in your fridge until all samples have been collected. Please post your samples back to us as soon as possible once all have been collected.

**Steps for packaging samples:**

1. Place all sample tubes back into the clear plastic bag they came in and seal it carefully.
2. Place sealed plastic bag into biohazard bag. Into this bag, place the white absorbent pad. Seal bag by removing adhesive strip.
3. Once you have assembled the postage box, place the biohazard bag inside. The box will already be stamped and marked with our address.
4. Place adhesive seal sticker in blue space marked on the outside of the box.
5. Place in any local post box.

Biohazard bag: Absorbent pad: Postage box: Seal sticker:


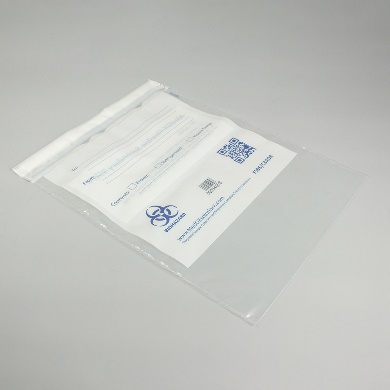

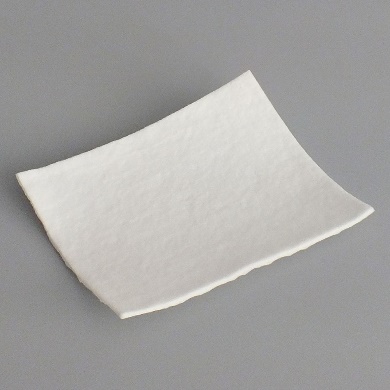

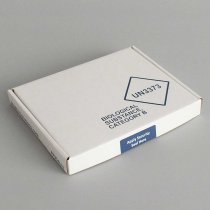

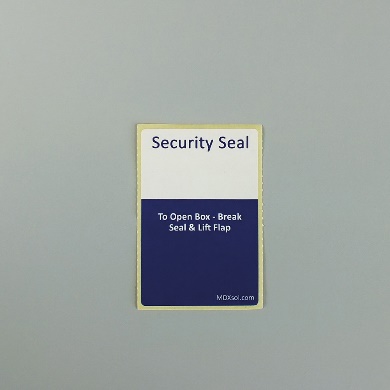


**Please post this completed booklet back to us separately from your samples, in the white stamped addressed envelope provided. PLEASE POST BOTH BOX AND ENVELOPE AT THE SAME TIME.**

Please remember to make a note of the date you will post the samples at the bottom of this page prior to sealing the envelope. If you are then unable to post on the date specified, please email the study team on **shaperpndo@kcl.ac.uk** to advise us of the correct date of postage.

**Date posted: ____ / ____ / ______**

**[OFFICE USE]**

**Date received: ____ / ____ / ______ Date stored: ____ / ____ / ______**

**How to collect the saliva samples (ADULT)**

|  | 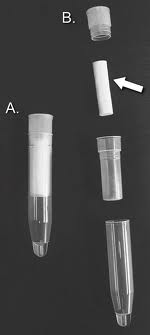 | Lid |
| --- | --- | --- |
|  |  | Swab |
|  |  |  |
| Salivette with swab |  | Inner tube |
|  |  | Outer tube |

|  | Take care to find the salivette tube marked with the appropriate time. |  |
| --- | --- | --- |
|  | Carefully remove the lid (the part on the end with ridges on). |  |
|  | Tip the swab into the lid and use this to place the swab in your mouth. Do not touch the swab with your fingers. |   **Swab**  **Lid** |
|  | Keep the swab in place for 1-2 minutes to ensure that it is saturated. |  |
|  | Take the swab out of your mouth with the help of the lid (so you are not touching the swab with your fingers). |   **Swab with saliva** |
|  | Carefully tip the swab into the salivette tube without touching it with your fingers. |  |
|  | Replace the lid firmly. |  |
|  | Store the samples in your fridge. |  |

**How to collect the saliva samples FROM YOUR BABY**

| Swab storage tube: | Salimetrics children’s swab: |
| --- | --- |
| 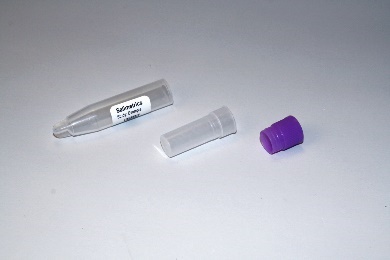 | 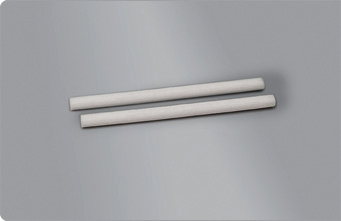 |

| 1. | Please wash and dry your hands carefully or wear gloves. | |
| --- | --- | --- |
| 2. | Take care to find the tube marked with the appropriate time. | |
| 3. | Take the swab, trying to avoid touching the swab with your fingers as much as possible. Carefully follow the instructions and diagram. | |
| 4. | Securely hold one end of the swab and place the other end under the child’s tongue. Reintroduce the swab as necessary for a total of 60-90 seconds. | |
| 5.  6. | Place the saturated swab directly into the swab storage tube, by folding, as shown in the diagram on the right.  Place the samples in a fridge until ready to post back. |  |
